# Malignancies in children and young adults on etanercept: summary of cases from clinical trials and post marketing reports

**DOI:** 10.1186/1546-0096-11-35

**Published:** 2013-10-02

**Authors:** Michele Hooper, Deborah Wenkert, Bojena Bitman, Virgil C Dias, Yessenia Bartley

**Affiliations:** 1 Amgen Inc., One Amgen Center Drive, Thousand Oaks, CA, USA; 2 Assent Consulting, Solana Beach, CA, USA

**Keywords:** Etanercept, Malignancy, Children, Young adults, TNF blocker, Juvenile idiopathic arthritis, Juvenile rheumatoid arthritis, Biologic disease-modifying antirheumatic drug

## Abstract

**Background:**

Malignancy risk may be increased in chronic inflammatory conditions that are mediated by tumor necrosis factor (TNF), such as juvenile idiopathic arthritis (JIA), but the role of TNF in human cancer biology is unclear. In response to a 2011 United States Food & Drug Administration requirement of TNF blocker manufacturers, we evaluated reporting rates of all malignancies in patients ≤30 years old who received the TNF blocker etanercept.

**Methods:**

All malignancies in etanercept-exposed patients aged ≤30 years from the Amgen clinical trial database (CTD) and postmarketing global safety database (PMD) were reviewed. PMD reporting rates were generated using exposure information based on commercial sources. Age-specific incidence rates of malignancy for the general US population were generated from the Surveillance Epidemiology and End Results (SEER) database v7.0.9.

**Results:**

There were 2 malignancies in the CTD: 1 each in etanercept and placebo/comparator arms (both in patients 18–30 years old). Postmarketing etanercept exposure was 231,404 patient-years (62,379 patient-years in patients 0–17 years; 168,485 patient-years in patients 18–30 years). Reporting rates of malignancy per 100,000 patient-years in the PMD and incidence rates in SEER were 32.0 and 15.9, respectively, for patients 0–17 years and 46.9 and 42.1 for patients 18–30 years old. Reporting rates were higher than SEER incidence rates for Hodgkin lymphoma in the 0-17 years age group. PMD reporting rates per 100,000 patient-years and SEER incidence rates per 100,000 person-years for Hodgkin lymphoma were 9.54 and 0.9, respectively, for patients 0–17 years and 1.8 and 4.2 for patients 18–30 years old. There were ≥5 cases of leukemia, lymphoma, melanoma, thyroid, and cervical cancers. Leukemia, non-Hodgkin lymphoma, melanoma, thyroid cancer, and cervical cancer rates were similar in the PMD and SEER.

**Conclusions:**

Overall PMD malignancy reporting rates in etanercept-treated patients 0–17 years appeared higher than incidence rates in SEER, attributable to rates of Hodgkin lymphoma. Comparison to patients with similar burden of disease cannot be made; JIA, particularly very active disease, may be a risk factor for lymphoma. No increased malignancy reporting rate in the PMD relative to SEER was observed in the young-adult age group.

## Background

The role of tumor necrosis factor (TNF) in inflammatory diseases such as rheumatoid arthritis (RA), juvenile idiopathic arthritis (JIA), and psoriasis is well established, as is the benefit of TNF inhibition in the treatment of these disorders [1]. TNF is key to natural killer cell-mediated destruction of tumor cells in mice [2]. The role of TNF in human cancer biology is less clear and it has been hypothesized that TNF inhibition may increase the risk of malignancies [3], particularly with prolonged, continuous exposure to TNF blockade [4]. An increased risk of skin cancers, including nonmelanoma skin cancer and melanoma, has been reported with the use of TNF blockers [5-7].

Adult patients with rheumatic diseases, especially those with severe disease, appear to have an inherently greater risk of lymphoma [8-11]. Chronic inflammation and autoimmune properties of these diseases are postulated to contribute to an increased risk of malignancy [11,12]. Although TNF has been reported to have anticancer properties, it has been suggested that TNF may also promote cancer development and progression through cellular transformation, tumor promotion, proliferation, invasion, angiogenesis, and metastasis [13].

Etanercept, a TNF receptor:Fc fusion protein, reversibly binds to and blocks the function of TNF [14]. In children, etanercept is approved in 100 countries and marketed in 108 countries for the treatment of JIA (starting in 1999) and for pediatric psoriasis in Europe (starting in 2009). In adults, etanercept is indicated for reducing signs and symptoms, inhibiting the progression of structural damage, and improving physical function in patients with moderately to severely active RA or active psoriatic arthritis; for reducing the signs and symptoms in patients with active ankylosing spondylitis; and for the treatment of chronic moderate to severe plaque psoriasis in patients who are candidates for systemic therapy or phototherapy.

Postmarketing data from the US Food and Drug Administration (FDA) Adverse Event Reporting System (AERS) regarding occurrences of malignancy in children in whom etanercept, infliximab, or adalimumab therapy was initiated between the ages of 0 and 18 years suggested an association between malignancies and TNF blockers [15]. The AERS data showed a 2.4-fold increased reporting rate of lymphoproliferative malignancies with etanercept treatment in children aged 0 to 18 years old relative to the general population as reported by the Surveillance, Epidemiology and End Results (SEER) database [15]. There was no increase in malignancies overall with etanercept. No clear causal relationship could be established since the cases were confounded by the underlying risk of malignancy in patients with autoimmune disease and the use of concomitant immunosuppressant therapies [15]. In a prior study using data from the Amgen clinical trials database (CTD) and the Amgen postmarketing global safety database (PMD) in children and adolescents aged 4 to 17 years, a total of 18 potential malignancies from 1998 to August 2009 were identified, including 4 cases of leukemia, 7 cases of lymphoma, and 7 solid tumors [16]. The results of that analysis did not suggest an association between etanercept use and malignancy overall, although there was a 3.8-fold increased reporting rate of lymphoma.

Both the published background rates of malignancy in patients with JIA as well as the rates in those treated with TNF blockers have varied widely. In a cohort of JIA patients from the Swedish Patient Register, there was a 4.2-fold increased risk for lymphoproliferative malignancy since 1987 and a 2.3-fold increase in overall malignancies in biologic-naïve JIA patients versus the general pediatric population [17]. Results from a US commercial claims database showed a 4-fold increased risk of malignancy in patients with JIA compared with the general pediatric population [18]. Conversely, data from three Canadian registries showed no increased risk of malignancy in patients with JIA [19]. In the US Medicaid database, a 4.4-fold higher risk of malignancy was reported in children aged 0 to < 16 years with JIA compared with children with asthma or attention deficit hyperactivity disorder [20]. No increased risk of malignancy was seen with the use of TNF blockers [20].

In 2009, the FDA required box warnings on the labels of TNF blocker medications to alert patients that lymphoma and other malignancies, some fatal, have been reported in children and adolescent patients treated with TNF blockers. In 2011, the FDA required pharmaceutical industry sponsors of TNF blockers to initiate a 10-year postmarketing commitment to expedite reporting of all global cases of malignancies in children and young adults exposed to TNF blockers and to report annually on their experience to date. We report the cumulative global reporting rates of all malignancies in children and young adults receiving etanercept through November 2011.

## Methods

### Data sources

Per the FDA postmarketing commitment requirements, patients up to and including the age of 30 years were included in the analysis. Data were obtained from the Amgen CTD and the Amgen PMD. Data in the CTD represent all subjects who participated in clinical trials of etanercept. Data in the PMD represent postmarketing and registry cases, but not cases reported in clinical trials. All cases of possible malignancies in the PMD were included in rate calculations, regardless of confounders or lack of histologic confirmation. The only cases that were excluded from the analysis were recurrent tumors when the primary tumor had occurred prior to the patient’s exposure to etanercept.

### Analyses

Exposure-adjusted reporting rates for patients in etanercept (with or without concomitant disease-modifying antirheumatic drugs) and placebo/comparator arms in the CTD were evaluated. Exposure data used to calculate the PMD reporting rates were derived from commercial sources and were based on the total amount (mg) of etanercept dispensed from November 1998 through December 2011 and assumed a dose of 50 mg/week. Patient-years (pt-yrs) of exposure were calculated as the total mg of etanercept dispensed divided by 52 weeks. The age distribution was derived from US market research and was extrapolated to the global market. Confidence intervals (CIs) were not calculated for the PMD rates because etanercept exposure data are estimates.

Exposure-adjusted reporting rates for malignancies overall and malignancies with 5 or more cases were generated, as were age-matched incidence rates for the general US population in the SEER database. The SEER database provides incidence rates for malignancies in the general US population with the exception of in situ cancers and nonmelanoma skin cancer. Age-specific incidence rates were generated from the SEER database v7.0.9.

## Results

### Malignancy rates in double-blinded clinical trials of etanercept

Two malignancies were reported in the CTD, including one case each in etanercept and placebo/comparator arms. Renal and urinary tract neoplasm were reported in a subject with psoriasis who received placebo and Hodgkin lymphoma was reported in a subject with RA who received etanercept. Both of these occurred in the 18–30 years age group.

### Malignancy reporting rates in the PMD and incidence rates in SEER

There were 103 malignancy reports in children and young adults ≤ 30 years of age: 79 in the 18–30 years age group, 19 in the 0–17 years age group, and 5 of unknown exact age but determined to be ≤ 30 years old, as shown by indication in Table 1. Twenty of the patients had JIA (12 of the 19 cases in those aged 0–17 years; 8 of the 79 cases in those aged 18–30 years). The estimated cumulative global exposure to etanercept for patients from 0 (ie, birth) to 30 years of age was 231,404 pt-yrs, including 62,379 pt-yrs for patients 0–17 years of age and 168,485 pt-yrs for patients 18–30 years of age. The reporting rate per 100,000 pt-yrs of all malignancies in the PMD was 32.0 for patients 0–17 years of age, and 46.9 for patients 18–30 years of age (Table 2). The incidence rate of all malignancies per 100,000 person-years in SEER was 15.9 for persons 0–17 years of age and 42.1 for persons 18–30 years of age.

**Table 1:** **Malignancy reports by TNF blocker indication for patients ≤ 30 years old in the PMD**^**a**^

**Age group, years**	**JIA**	**RA**	**AS**	**PsA**	**Psoriasis**	**Other**	**Not reported**	**Total**
0 to <6	1	0	0	0	0	0	1	2
6 to <12	4	0	0	0	0	0	0	4
12 to <18	7	0	0	0	0	2	4	13
18 to 30	8	29	14	5	17	2	4	79
Unknown	0	0	2	0	3	0	0	5
Total	20	29	16	5	20	4	9	103

^a^Data represent number of cases reported.

AS, ankylosing spondylitis; JIA, juvenile idiopathic arthritis; PMD, postmarketing database; PsA, psoriatic arthritis; RA, rheumatoid arthritis; TNF, tumor necrosis factor.

**Table 2:** Overall malignancy reports and rates in the PMD and SEER

	**PMD**		**SEER**
**Age group,**^**a **^**years**	**Reports, n**	**Reports per 100,000 pt-yrs**	**Incidence rate per 100,000 pt-yrs (95% CI)**
0 to 17	19	32.0	15.9 (15.7-16.0)
18 to 30	79	46.9	42.1 (41.7-42.4)

^a^5 reports not included because age was unknown.

CI, confidence interval; PMD, postmarketing database; pt-yrs, patient-years; SEER, Surveillance, Epidemiology and End Results.

### Malignancies with ≥ 5 reports in the PMD

At least 5 reports each of melanoma (7 cases, all in patients 18–30 years old), thyroid cancer (6 cases: 1 case in a patient 0–17 years old and 5 cases in patients 18–30 years old), and cancer of the cervix excluding carcinoma in situ (6 cases in patients 18–30 years old) were recorded in the PMD. The reporting rates for melanoma, thyroid cancer, and cervical cancer approximated the SEER incidence rates for the 0–17 years and 18–30 years age groups. Eight cases of leukemia were reported (Table 3). Reporting rates were higher than SEER incidence rates for Hodgkin lymphoma in the 0–17 years age group (Table 3). There were 23 cases of lymphoma reported, including 9 cases of Hodgkin lymphoma, 7 cases of non-Hodgkin lymphoma (2 cases of diffuse large B-cell lymphoma, 2 cases of mucosa-associated lymphoid tissue [MALT] lymphoma, 1 case of Burkitts lymphoma, 1 case of B-cell unknown lymphoma, and 1 case of mycosis fungoides), and 7 cases of lymphoma not otherwise specified.

**Table 3:** Leukemia and lymphoma reporting rates in the PMD and incidence rates in SEER

	**0 to 11 years**		**12 to 17 years**		**18 to 30 years**	
	**n**	**Rate**^**a**^	**n**	**Rate**^**a**^	**n**	**Rate**^**a**^
Leukemia						
PMD	3	12.1	0	0	5	3.0
SEER		5.4		3.2		2.6
Lymphocytic leukemia Leukemia						
PMD	2	8.1	0	0	2	1.2
SEER		4.4		2.1		0.9
Myeloid leukemia						
PMD	0	0	0	0	3	1.8
SEER		0.9		1.0		1.5
Leukemia not otherwise specified						
PMD	1	4.0	0	0	0	0
Lymphoid neoplasm						
PMD	3	12.1	5	13.1	15	8.9
SEER		5.5		5.7		8.0
Hodgkin lymphoma						
PMD	3	12.1	3	7.9	3	1.8
SEER		0.4		2.0		4.2
Non-Hodgkin lymphoma						
PMD	0	0	1	2.6	6	3.6
SEER		5.2		3.6		3.7
Lymphoid not otherwise specified						
PMD	0	0	1	2.6	6	3.6

^a^Rates represent reports per 100,000 patient-years in the PMD and cases per 100,000 person-years in SEER.

PMD, postmarketing database; SEER, Surveillance, Epidemiology and End Results.

## Discussion

The emerging literature suggests that there may be an increased risk of malignancy in children with JIA that is unrelated to treatment with TNF blockers; however, the data are not consistent and are derived from a mix of patient populations [17-20]. Additionally, symptoms of some malignancies may mimic the symptoms of JIA and artificially inflate the rates reported for JIA. The paucity of detail from postmarketing reports makes this difficult to determine. Comparison of the reporting rates of malignancy in etanercept-exposed children and young adults with those reported in the literature should be done with caution because these are different patient populations. Comparison to SEER rates also requires caution as SEER represents the general population rather than a disease population and reports incidence rather than reporting rates. Nevertheless, relative to incidence rates in the general population as reported in the SEER database, reporting rates for malignancies in the PMD were higher in the 0–17 years age group but not the 18–30 years age group. The only tumor type that appeared to be increased was Hodgkin lymphoma.

Lymphomas constitute approximately 10% to 15% of all cancers in children and are the third most common pediatric malignancy [21]. Hodgkin lymphoma, the most common form, represents nearly 50% of all cases of lymphoma [21]. Non-Hodgkin lymphoma accounts for approximately 5% to 10% of all childhood cancers and typically occurs in children > 5 years old [21,22]. Eight of 9 cases of Hodgkin lymphoma observed in the PMD reported concomitant or prior immunosuppressants, including 5 who received multiple agents (including cytotoxic therapies, other biologics, cyclosporine, and thalidomide) and 3 who received methotrexate only.

Acute leukemia, including acute lymphoblastic leukemia (ALL), is the most common cancer in children, accounting for a third of all malignancies [22]. Approximately 80% of cases of childhood leukemia in patients younger than 15 years are ALL [23], with a peak incidence from 3 to 5 years of age [22]. Of the eight cases of leukemia reported in our analysis, four cases (3 cases of ALL and 1 case of chronic myelogenous leukemia [CML]) had ≤ 3 months exposure to etanercept, making it very unlikely that etanercept had a causal relationship. Baseline hematologic abnormalities in some of the ALL cases suggested that they were possibly leukemia presenting as a rheumatologic condition. The report of a diagnosis of leukemia not otherwise specified was based on marrow hypercellularity as seen on magnetic resonance imaging in a 10-year-old patient with seronegative enthesopathy syndrome who was classified as a patient with JIA, with no additional clinical factors reported. Melanoma accounts for 7% of all cancers among individuals 15 to 19 years old and 1.2% of all cancers among individuals less than 15 years of age [24]. Results from a recent analysis of data from a Swedish population-based prospective cohort study (ARTIS) suggested that adults with RA who have been treated with a TNF blocker have a 50% increased relative risk of invasive melanoma relative to RA patients who have not been treated with a TNF blocker [25]. Reporting rates for melanoma in the PMD were comparable to the incidence rates in SEER.

In adults with plaque psoriasis and psoriatic arthritis, a systematic review and meta-analysis of randomized, placebo-controlled studies of the TNF blockers etanercept, infliximab, adalimumab, golimumab, and certolizumab suggested no statistically significant evidence of an increased risk of cancer with these treatments. The odds ratio for all malignancies was 1.48 (95% confidence interval [CI]: 0.71–3.09) [26]. A recent meta-analysis of 7 prospective studies on all-site malignancies reported a lack of evidence that patients with RA treated with TNF blockers are at increased risk of malignancies when compared with nonexposed RA patients (pooled relative risk = 0.95, 95% CI: 0.85–1.05) [6].

In some tumors associated with chronic inflammation, TNF inhibition may be protective, such as follicular lymphoma [27], and as demonstrated in a mouse model of dextran sulfate sodium and azoxymethase-induced colitis and colon cancer [28]. Conversely, TNF may be an important inhibitor in the melanoma microenvironment [29].

A limitation of this report is the lack of a suitable comparator to determine the potential effect of etanercept therapy on the risk of malignancy. Incidence rates in SEER are based on confirmed cases from the general population (not just people with chronic inflammation). The SEER database also has disproportionally fewer numbers of younger patients relative to the PMD; therefore, a small number of cases in the younger age group in the PMD leads to artificially higher reporting rates relative to SEER incidence rates. Patients with all etanercept indications (JIA, RA, ankylosing spondylitis, psoriatic arthritis, and psoriasis) were included in the analysis and thus the observed rates may not be generalizable across indications. An additional limitation is the lack of histologic documentation of malignancy in many cases in the PMD, which may result in over-reporting of some malignancies. Under-reporting of adverse events is common in registries and postmarketing surveillance programs, although this might be less likely with pediatric cancers.

## Conclusions

In summary, overall malignancy reporting rates for pediatric patients receiving etanercept appeared to be higher in the 0–17 years age group compared with incidence rates from a similarly aged pediatric population, but not in young adults aged 18–30 years. The increase in overall reporting rate appears to be due to the increase in reports of Hodgkin lymphoma. Reporting rates of Hodgkin lymphoma in etanercept-exposed patients ages 0–17 years were higher than incidence rates reported in SEER. Of the five most commonly reported malignancies in the PMD, reporting rates for melanoma, thyroid cancers, cervical cancer, and leukemia appeared to be similar to incidence rates reported in SEER. Although these comparisons cannot establish cause and effect, they do provide the clinician with estimates of the malignancy reporting rates in a treated population. Although the number of cases is too small to draw meaningful conclusions about some specific tumor types, these data do not suggest a relationship between etanercept use and the development of solid tumors. Amgen will continue to monitor malignancies and report the malignancy experience with etanercept-exposed children and young adults.

## Competing interests

MH, DW, BB, and VD are employees and shareholders of Amgen Inc. YB is an employee of Assent Consulting, which received support for this study from Amgen Inc.

## Authors’ contributions

MH, DW, BB, and VD contributed to the study design and analysis and interpretation of the data. YB contributed to the analysis of the data. All authors contributed to the writing and revision of the manuscript and all authors approved the final draft of the manuscript.
